# Verrucous Sarcoidosis: A Rare Clinical Presentation of Sarcoidosis

**DOI:** 10.7759/cureus.15175

**Published:** 2021-05-22

**Authors:** Morgan E Sussman, Bobak T Pousti, Shoshana K Grossman, Jason B Lee, Sylvia Hsu

**Affiliations:** 1 Department of Dermatology, Temple University, Lewis Katz School of Medicine, Philadelphia, USA; 2 Department of Dermatology, University of California San Diego School of Medicine, San Diego, USA; 3 Department of Dermatology and Cutaneous Biology, Thomas Jefferson University, Philadelphia, USA

**Keywords:** verrucous sarcoidosis, cutaneous sarcoidosis, adalimumab, pulmonary sarcoidosis, inflammatory dermatoses

## Abstract

Verrucous sarcoidosis is a rare cutaneous variant of sarcoidosis, an inflammatory disease characterized by non-caseating granulomas that primarily involves the lungs. The current literature on verrucous sarcoidosis is limited, with the majority of lesions presenting on the lower extremities of African American males. Here, we present two cases that highlight the unique manifestations of this uncommon cutaneous entity. The first case involves a middle-aged Hispanic woman with lesions on her arms and face, and the second case involves a middle-aged African American woman with sole facial involvement. A multi-disciplinary approach to diagnosis and treatment is required as verrucous sarcoidosis can present with clinical and histopathological features indistinguishable from infectious etiologies and has an association with pulmonary sarcoidosis. Adalimumab has demonstrated success in the treatment of verrucous sarcoidosis.

## Introduction

Sarcoidosis is a chronic, inflammatory disease that manifests across multiple organ systems. This condition is found more commonly in women, particularly those of African descent. Compared to their Caucasian counterparts, African Americans tend to present earlier and with more acute manifestations [1]. Although the majority of cases impact the pulmonary system, this granulomatous condition can present with various extrapulmonary manifestations [2].

After the lungs, the skin is the next most commonly affected organ. Cutaneous involvement is often an early finding and is present in up to 35% of cases [3]. A rare cutaneous variant of this condition is referred to as verrucous sarcoidosis. The current literature on this cutaneous manifestation is limited to 17 cases, with the majority of lesions presenting on the lower extremities and in African American patients [3-5]. Patients with this verrucous variant often have underlying pulmonary sarcoidosis [5-7], necessitating evaluation for respiratory infiltration [8]. Verrucous lesions may occur alone or in conjunction with ulcerative, papillomatous, or more typical papular lesions [5-7]. Here, we present two cases of verrucous sarcoidosis.

## Case presentation

Case 1

A 51-year-old Hispanic woman presented with a two-year history of progressive pruritic plaques on her face, upper extremities, and trunk. She was diagnosed with pulmonary sarcoidosis in 2005. Five years later, she developed cutaneous sarcoidosis, confirmed by a skin biopsy showing non-caseating granulomas. Her systemic sarcoidosis had been treated with chronic prednisone up to 40 mg daily. Two months prior to presentation, the patient’s pulmonologist initiated her on methotrexate 7.5 mg daily as adjunctive therapy to the prednisone. Neither treatment had improved her skin eruption.

**Figure 1:**
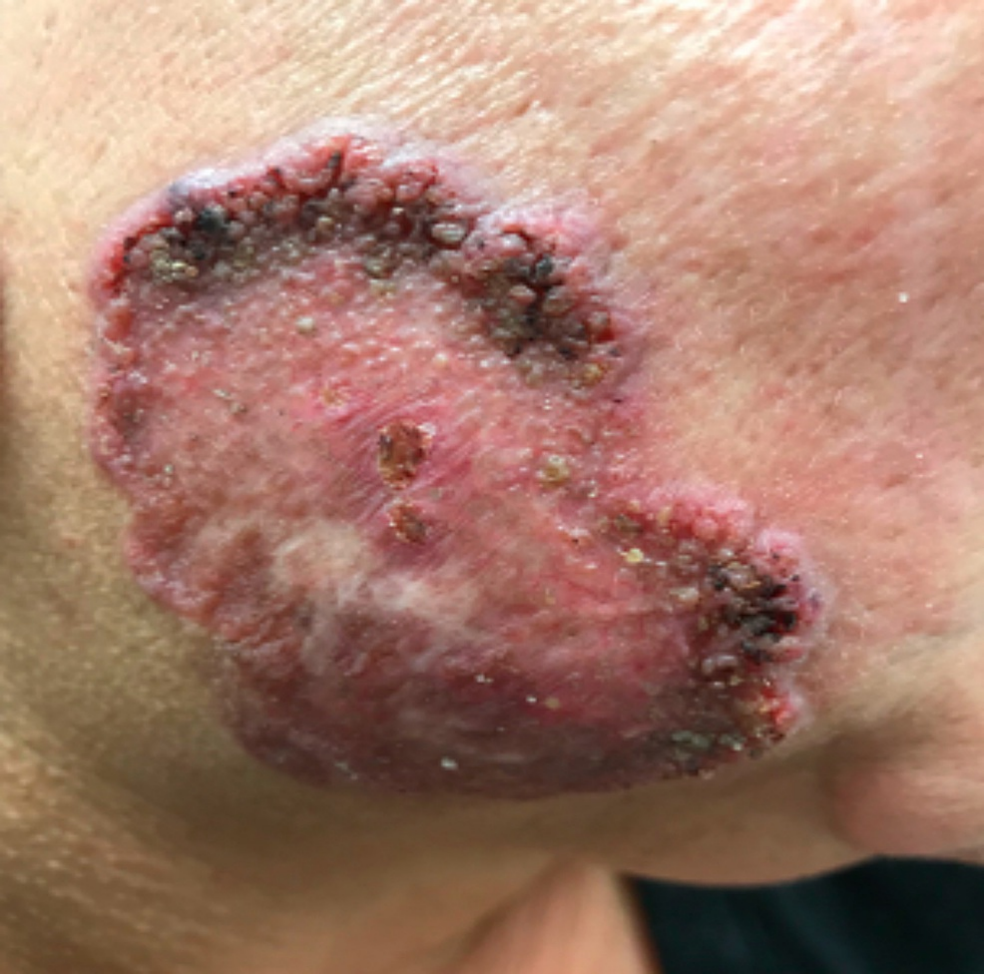
Large annular, hyperkeratotic, verrucous erythematous plaque on the cheek.

On physical examination, there were hyperkeratotic, annular, erythematous plaques with heaped-up borders on her arms and face (Figure 1), as well as erythematous, non-scaly plaques on her arms and back.

**Figure 2:**
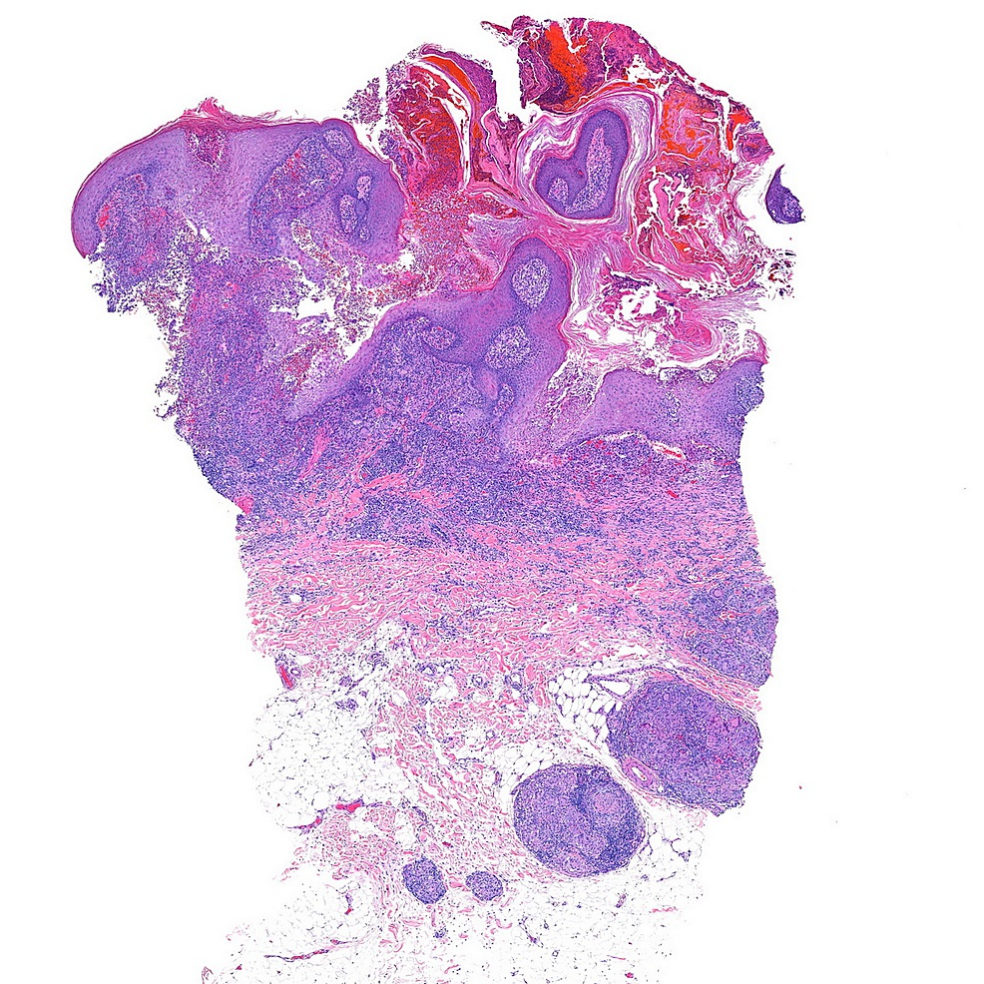
Verrucous pseudoepitheliomatous hyperplasia of the epidermis and sarcoidal nodular granulomatous infiltrate throughout the dermis (H&E, 40×). H&E: hematoxylin and eosin

**Figure 3:**
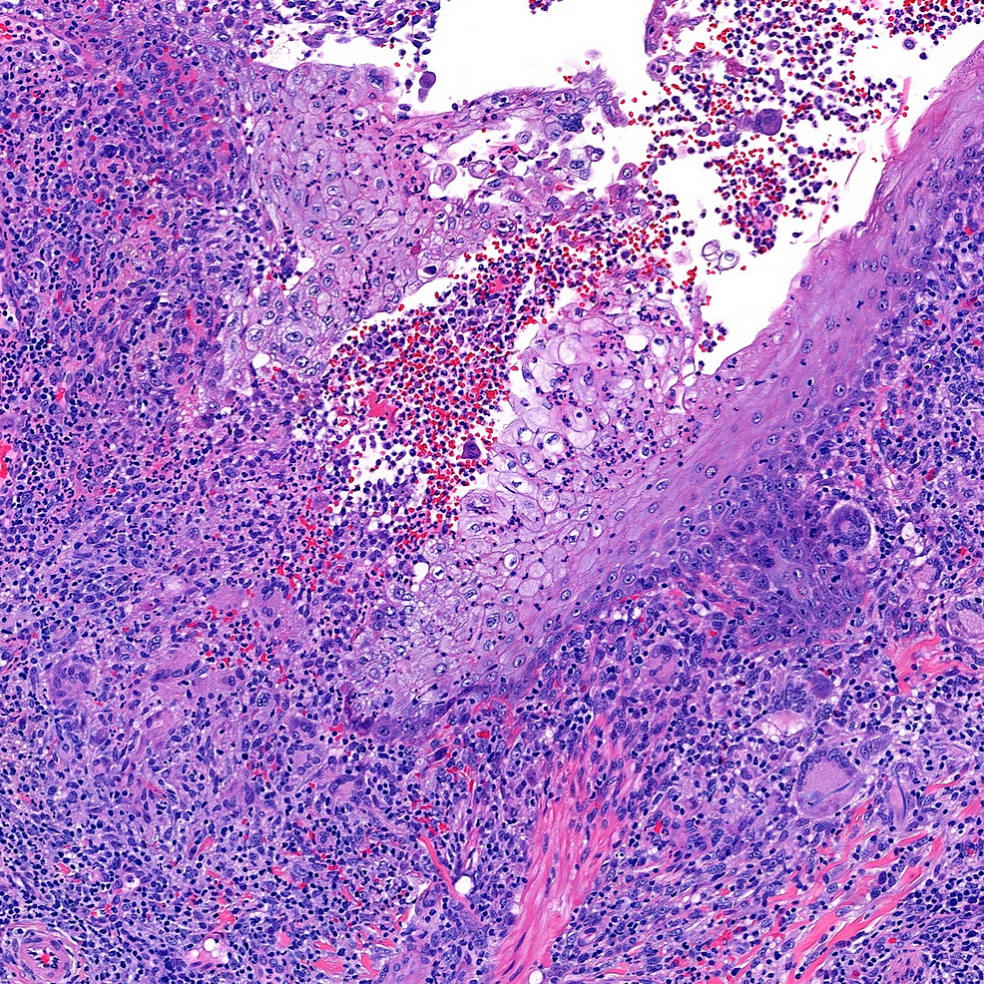
Suppurative granulomatous inflammatory infiltrate due to the disruption of the epidermis (H&E, 200×). H&E: hematoxylin and eosin

A total of four punch biopsies were performed, two from the verrucous lesion of the face and two from the non-verrucous lesions. Biopsies from the non-verrucous lesions demonstrated nodular sarcoidal granulomas. Two biopsies of the verrucous plaque on the right cheek showed striking verrucous hyperplasia with suppurative granulomatous inflammation in the superficial dermis and nodular sarcoidal granulomas in the deep dermis (Figures 2, 3). Periodic acid-Schiff, Gomori’s methenamine silver, acid-fast bacilli (AFB), and Fite stains failed to reveal organisms. Tissue cultures for bacteria and fungus failed to isolate organisms. An interferon-gamma release assay for tuberculosis was negative.

After carefully excluding an infectious etiology, cutaneous verrucous expression of sarcoidosis was established. The patient was started on adalimumab with marked improvement after nine months of therapy and continued improvement after 21 months of therapy.

Case 2

**Figure 4:**
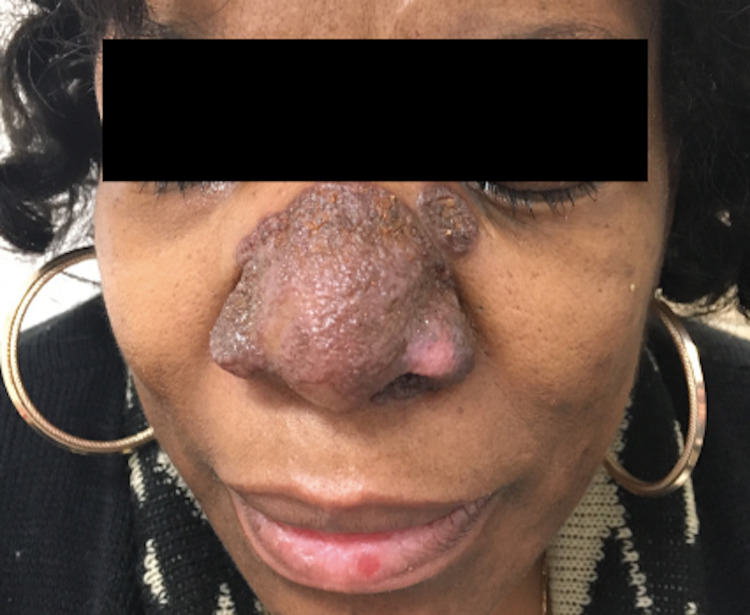
Well-defined, red-brown verrucous plaque on the nose.

A 60-year-old African American woman presented with a 10-month history of plaques on her nose and preauricular area (Figure 4). Her past medical history was significant for pulmonary and sinonasal sarcoidosis diagnosed in 2000. She was taking methotrexate 12.5 mg weekly as prescribed by her pulmonologist. On physical examination, the patient was noted to have well-defined, red-brown-colored verrucous plaques on her nose and preauricular area.

A 4-mm punch biopsy of the nose revealed marked pseudoepitheliomatous hyperplasia with underlying nodular sarcoidal granulomas in the dermis, consistent with sarcoidosis. An AFB stain revealed no organisms.

The patient was treated with intralesional triamcinolone. No significant improvement was noted after one month, and she was started on 0.05% clobetasol ointment. Four months following clobetasol initiation, the patient had marked improvement. She also continued her weekly methotrexate.

## Discussion

Verrucous sarcoidosis is an exceedingly rare manifestation of cutaneous sarcoidosis. Most of these cases involve the lower extremities of African American men [9]. Furthermore, this particular variant is associated with long-standing pulmonary disease.

Our cases represent unique manifestations of this uncommon entity. First, our patients are both females rather than males. Additionally, our first patient is Hispanic instead of African American. There are only four other reported cases of verrucous sarcoidosis in a non-African American patient [4,5,10,11]. Neither of our patients had lesions in the classic distribution of the lower extremities, but rather lesions at the face, trunk, and upper extremities. Of note, both of our patients experienced pulmonary sarcoidosis prior to their cutaneous manifestations, a reported association in those with verrucous lesions [5,7].

Sarcoidosis has often been referred to as the “great imitator” of other skin diseases [12]. This is evident in the verrucous presentation of sarcoidosis, which represents a potential diagnostic pitfall. Verrucous sarcoidosis, similar to the verrucous expression of other inflammatory diseases, may simulate squamous cell carcinoma and infectious diseases associated with pseudoepitheliomatous hyperplasia, such as deep fungal and atypical mycobacterial infections. In both of our cases, striking pseudoepitheliomatous hyperplasia that was indistinguishable from an infectious etiology was present. In the first case, not only was pseudoepitheliomatous hyperplasia present but also suppurative granulomatous inflammation, requiring an extensive workup to exclude an infectious etiology. The suppurative inflammation was most likely due to a rupture of the epidermis. In contrast, the second case showed more classic nodular sarcoidal granulomas associated with pseudoepitheliomatous hyperplasia. Differentiating verrucous sarcoidosis from alternative pathologies requires both a strong clinical correlation and histopathologic analysis. Appropriate immunohistochemical staining, tissue cultures, serum analysis, and imaging may be required to rule out infectious etiologies and squamous cell carcinoma.

Initial treatment of cutaneous sarcoidosis can incorporate topical, intralesional, and systemic steroids [13]. Alternative immunomodulators, such as hydroxychloroquine and methotrexate, have also been used with positive results [14-17]. Recent literature suggests that the use of adalimumab can improve verrucous sarcoidosis. This human monoclonal antibody against tumor necrosis factor-alpha (TNF-α) has anecdotally been shown to yield 95% clearance of verrucous lesions [18]. The utilization of adalimumab for cutaneous sarcoidosis follows successful prior use of anti-TNF-α agents, such as infliximab, for more typical presentations of cutaneous sarcoidosis [19,20].

## Conclusions

In conclusion, verrucous sarcoidosis is a rare cutaneous variant of sarcoidosis that may present with clinical and histopathological findings that are indistinguishable from an infectious etiology, such as a deep fungal infection. As verrucous sarcoidosis has a high clinical association with pulmonary sarcoidosis, this rare expression of sarcoidosis should be managed with an interdisciplinary healthcare team. Treatment modalities include corticosteroid, immunomodulatory, and biologic therapies. Such lesions are often stubborn in response and may require a multi-faceted approach.

## References

[1] Judson MA, Boan AD, Lackland DT (2012). The clinical course of sarcoidosis: presentation, diagnosis, and treatment in a large white and black cohort in the United States. Sarcoidosis Vasc Diffuse Lung Dis.

[2] Baughman RP, Teirstein AS, Judson MA (2001). Clinical characteristics of patients in a case control study of sarcoidosis. Am J Respir Crit Care Med.

[3] Hudson AD, Klimas NK, Stetson CL (2018). Filiform verrucous sarcoidosis of the face: a warty report. J Cutan Med Surg.

[4] Siham M, Asmaa S, Nadia I, Laila B, Badr H, Karima S (2018). An unusual variant of cutaneous sarcoidosis. Clin Med Img Lib.

[5] DeFelice T, Fischer M, Kamino H, Cohen D, Latkowski JA (2012). Verrucous and macular sarcoidosis. Dermatol Online J.

[6] Smith HR, Black MM (2000). Verrucous cutaneous sarcoidosis. Clin Exp Dermatol.

[7] Koch LH, Mahoney MH, Pariser RJ (2010). Cutaneous sarcoidosis manifesting as extensive verrucous plaques. Int J Dermatol.

[8] Esteves TC, Aparicio G, Ferrer B, Garcia-Patos V (2015). Prognostic value of skin lesions in sarcoidosis: clinical and histopathological clues. Eur J Dermatol.

[9] Stockman DL, Rosenberg J, Bengana C, Suster S, Plaza JA (2013). Verrucous cutaneous sarcoidosis: case report and review of this unusual variant of cutaneous sarcoidosis. Am J Dermatopathol.

[10] Pezzetta S, Zarian H, Agostini C, Salmaso R, Alaibac M (2013). Verrucous sarcoidosis of the skin simulating squamous cell carcinoma. Sarcoidosis Vasc Diffuse Lung Dis.

[11] Hinojosa T, Lewis DJ, Sharghi KG (2017). Verrucous eyebrows: a cutaneous manifestation of a systemic disease. J Eur Acad Dermatol Venereol.

[12] Tchernev G (2006). Cutaneous sarcoidosis: the "great imitator": etiopathogenesis, morphology, differential diagnosis, and clinical management. Am J Clin Dermatol.

[13] Katta R (2002). Cutaneous sarcoidosis: a dermatologic masquerader. Am Fam Physician.

[14] Webster GF, Razsi LK, Sanchez M, Shupack JL (1991). Weekly low-dose methotrexate therapy for cutaneous sarcoidosis. J Am Acad Dermatol.

[15] Siltzbach LE, Teirstein AS (1964). Chloroquine therapy in 43 patients with intrathoracic and cutaneous sarcoidosis. Acta Med Scand Suppl.

[16] Morse SI, Cohn ZA, Hirsch JG, Schaeder RW (1961). The treatment of sarcoidosis with chloroquine. Am J Med.

[17] Veien NK, Brodthagen H (1977). Cutaneous sarcoidosis treated with methotrexate. Br J Dermatol.

[18] Pariser RJ, Paul J, Hirano S, Torosky C, Smith M (2013). A double-blind, randomized, placebo-controlled trial of adalimumab in the treatment of cutaneous sarcoidosis. J Am Acad Dermatol.

[19] Hagan CE, Offiah M, Brodell RT, Jackson JD (2018). Chronic verrucous sarcoidosis associated with human papillomavirus infection: Improvement with adalimumab. JAAD Case Rep.

[20] Haley H, Cantrell W, Smith K (2004). Infliximab therapy for sarcoidosis (lupus pernio). Br J Dermatol.

